# Aid after enrollment: Impacts of a statewide grant program at public two-year colleges

**DOI:** 10.1016/j.econedurev.2018.10.008

**Published:** 2018-12

**Authors:** Drew M. Anderson, Sara Goldrick-Rab

**Affiliations:** a RAND Corporation, 1776 Main St., Santa Monica, CA 90401, United States; b Temple University, 419 Ritter Annex, 1301 Cecil B. Moore Avenue, Philadelphia PA 19122, United States

## Abstract

Most students who begin at community colleges do not finish with a degree. The net price of college commonly shifts after enrollment, and there is little evidence on how these shifts affect two-year degree completion. This study provides evidence on the impacts of a private program that offered supplemental grant aid to students with demonstrated financial need, who were already enrolled at public two-year colleges in Wisconsin. An evaluation using a randomized control group and data from over 4,000 students in multiple cohorts, fails to confirm substantial increases in persistence or degree completion caused by the grant program. We highlight some features of implementation that added complexity and may have reduced the effectiveness of the grant program.

## Highlights

• Financial aid can buffer against unexpected changes after college enrollment.
• We study a randomly assigned supplement to the Pell Grant at two-year colleges.
• We do not detect substantial increases in college attainment from the added grants.
• Recent research points to complexity as a barrier which is as important as cost.
• This aid added complexity for some which may have reduced effectiveness on average.

## 1 Introduction

For most students, the price of college will change each year they stay enrolled. Changes in net price can come from shifts in tuition, living expenses, or financial aid. At the same time, changes in the net benefit of college can come from shifts in the labor market and the opportunity cost of foregone earnings. These changes occur at a high enough frequency to interrupt students seeking two-year degrees. Of nearly one million students who entered community colleges in fall 2008, 74% dropped out or stopped out without earning a degree (Shapiro, Dundar, Yuan, Harrell, & Wakhungu, 2014).

The high rate of dropout suggests there are important shifts in the benefit-cost calculation that occur after enrollment, and there is potential for financial interventions to increase the net benefit and decrease dropout. However financial aid comes with uncertainty of its own. Changes in aid can come from modifications to financial aid policy, or from shifts in the student's financial or academic qualifications for existing policies. This study evaluates the effects of a new financial aid program delivering aid after enrollment, and highlights both the aid and the additional complexity the program introduced.

The late 2000s saw large increases in spending on need-based financial aid from federal, state, and institutional sources, nearly doubling over a three-year period following the financial crisis of 2008 (U.S. Department of Education, 2015). At community colleges, the recession and tight budgets brought rising tuition and falling instructional spending (Barr & Turner, 2013). Median family income also fell, leaving families less equipped to pay tuition (Bricker et al., 2014). Compared to students at four-year universities, community college students received less state and institutional financial aid to offset these trends, and thus relied primarily on the federal Pell Grant (College Board, 2015). Pell Grant awards expanded on a per-recipient bases, with the average award growing by 30% in the three-year period following 2008 (U.S. Department of Education, 2015).

A key question is whether the growth in Pell Grants over this period prevented college dropout. This question can be challenging to answer because comparison groups are difficult to find for students benefiting from this national program, which underwent policy shifts coincident with shifts in many other economic factors. Recent studies of Pell Grant aid use regression discontinuity designs and focus on university students local to eligibility cutoffs (e.g. Denning, 2017a, Denning et al., 2017, Marx and Turner, 2018). This tight focus leaves gaps in our knowledge about this important public policy, including the treatment effect of Pell Grant aid for community college students, who as a group receive more Pell Grant dollars than public university students or private college students (U.S. Department of Education, 2015).

To address this question, our study exploits an exogenous shift in financial aid for Pell-eligible students. The privately funded Wisconsin Scholars Grant (WSG) offered $1,800 per year to students from low-income families, who were enrolled full-time at public technical colleges and two-year branch campuses of the public university system in Wisconsin. These students faced tuition charges of $3,300 to $4,600. The average student received $6,700 in grants (not including the WSG) and borrowed an additional $1,800 to cover tuition, fees, books, supplies, and living expenses. Typically about a quarter of these students dropped out or dropped to part-time after one semester. WSG arrived during the first semester, and was intended to meet financial need, lower borrowing, avert dropout, and support degree completion. Random assignment allows us to test for average effects of the WSG, and to examine whether it was more effective for students with greater financial need.

This study complements Goldrick-Rab et al. (2016), which analyzed the WSG's effects on university students, who received $3,500 per year from the program. That analysis found a positive effect on four-year degree completion, which is broadly consistent with earlier findings that enrolled university students benefit from additional need-based aid (Deming & Dynarski, 2010; Page and Scott-Clayton, 2016; Castleman & Long, 2016).

Overall this study cannot confirm a strong positive impact of the WSG on college attainment for two-year college students. Using a 95% confidence interval in our sample of over 4,000 students, our estimates rule out impacts larger than a 2.3 percentage-point increase in the rate of persistence to the second year of college per $1,000 of (yearly) aid offered, a smaller effect per dollar than has been estimated for university students. Considering increases in the rate of on-time degree completion per $1,000 of aid offered, our confidence intervals include the size of positive impacts estimated in Goldrick-Rab et al. (2016), but they also include zero impact.

We find suggestive evidence the WSG is more effective at supporting increased credit attainment among students with greater financial need. Also, students receiving the grant were less likely to laterally transfer to a different two-year college, but no more likely to transfer to a four-year university.

Recipients of the WSG applied the grant money to current expenses, and on average did not leave college with significantly less student loan debt. Within this sample, the majority of students do not borrow, and the vast majority of those who do borrow take the maximum amount offered to them. Therefore most students are at the corners of the borrowing space, making it empirically difficult to detect small changes in a student's desired borrowing amount. We estimate that the WSG did not affect either the decision to borrow or the decision of how much to borrow while enrolled, nor did it significantly affect overall debt through the channel of longer enrollment. Even though students face financial challenges, even insecurity in food and housing, they may avoid borrowing because they do not expect to be in a position to pay back loans, or because they are averse to borrowing (Boatman et al., 2017, Broton and Goldrick-Rab 2017).

Our estimates are consistent with a growing body of evidence showing that low-income community college students benefit more from guidance and services than from additional financial aid (Carruthers and Fox, 2016; Barrow et al., 2014; Baker, 2016, Barrow et al., 2014, Scott-Clayton, 2011, Scrivener et al., 2015). Several randomized studies have compared aid-only interventions against wraparound services, and found greater impacts of services (Angrist et al., 2009, Carrell and Sacerdote, 2017, Clotfelter et al., 2017, Evans et al., 2017).

The WSG not only lacks support services, it may have introduced additional complexity that was not offset by additional grant dollars. In these early years of operation for the grant program, about 25% of the students who were placed into the randomization pool by school officials were apparently not eligible for the WSG. When students in this group were randomly selected and contacted about their grant offers, they were unable to verify eligibility and were not awarded grants. They experienced worse enrollment outcomes than similar comparison group students who were neither offered nor awarded grants. Prior research shows that complexity of paperwork can inhibit access to financial aid and success in college (Dynarski & Scott-Clayton, 2006; Bettinger, Long, Oreopoulos, & Sanbonmatsu, 2012; Page and Scott-Clayton, 2016). We highlight an understudied aspect of complexity: that school officials must administer complex aid programs, which can develop and change constantly.

Improving the delivery of aid programs can have important consequences for students. The overall benefits of two-year degrees are substantial and growing (Belfield & Bailey, 2011). Additional college credits can lead to substantial increases in employment and earnings, with completed degrees and certificates in highly demanded fields particularly valuable (Marcotte et al., 2005, Dadgar and Trimble, 2015, Xu and Trimble, 2016, Carruthers and Sanford, 2018, Stevens et al., 2018). It is therefore important to identify what could be improved, not only what works, when evaluating policies and programs to increase college completion.

The remainder of the paper expands on the implementation of the WSG and its potential impacts, our research design, and our empirical results. We conclude with a discussion of our findings in the context of current policies affecting the price of college after enrollment.

## 2 The Wisconsin Scholars Grant (WSG)

The WSG is a privately funded grant program seeking to offset college costs for low-income Wisconsin families, and increase college completion. This section describes the operation of the WSG and its random assignment process, the program's goals and potential effects on students, and our research design to test for its effectiveness at reaching those goals.

### 2.1 Operation of the WSG

The Fund for Wisconsin Scholars operates the WSG in cooperation with school and state officials. Students were selected for the WSG randomization pool based on a set of criteria observable on their Free Application for Federal Student Aid (FAFSA) and their college admissions records. The main criterion was Pell Grant eligibility. For the 2008–09 school year, the first year the program operated, Pell eligibility corresponded roughly to a family income below $50,000, earned in the 2007 calendar year. In addition to being Pell-eligible, students had to have recently graduated from a public high school in Wisconsin (within the past three years), be enrolled for the first time at a public two-year college in state and registered for at least 12 credit hours (full-time status), and have demonstrated financial need (their other grant aid plus their Expected Family Contribution calculated on the FAFSA could not exceed their total costs including living expenses).

The Fund set a grant size intended to be transformational for students who received it. The grant provided $1,800 per year, and if students transferred to a public university in the state, the grant increased to $3,500 per year. At Wisconsin's two-year colleges, the WSG amounted to around half of tuition and fees, though the average student in the WSG target population already had tuition and fees covered by other grant aid. The grant therefore was intended to offset borrowing or living costs that could act as barriers to continued enrollment and graduation.

Combining the desired individual grant amount, the size of the target population, and its endowed resources, the Fund for Wisconsin Scholars could not fully fund every eligible student. With input from our research team, the Fund decided to award grants by lottery among the target population. This design would continue in perpetuity and allow for evaluation of program effectiveness. The administration of the lottery and the delivery of awards relied on actions by the Fund, financial aid officials, and students themselves.

Placement into the randomization pool was done by administrators without students’ knowledge, after enrollment in September of their first year. As there were no additional requirements of students to enter the randomization, students were not notified about the WSG at application time. Non-selected (control group) students were never contacted by the Fund. Each year, roughly 1,500 students entered the randomization pool across 17 colleges. Each year, roughly 600 of these students were selected to receive grant offers.

Throughout our analysis we focus on the group randomly selected to receive WSG *offers*. However it is important to also discuss who received WSG *awards*. A student could only enter the randomization pool once, and selection remained “on” for the duration of the student's future academic career, but there were additional requirements to receive aid both initially and in subsequent years.

Once selected, students received a letter in October, and had to sign and return a form to verify their eligibility and receive the grant. Of the initially offered students, only 80% of these ever received the grant, indicating a substantial portion did not receive the notification, did not return it, or were deemed ineligible. A significant portion of the initial non-receipt was driven by students who were ineligible being placed into the randomization pool by school officials, mainly in the first cohort of the program at technical colleges.

Students could continue to receive the WSG for up to ten semesters if they continued to re-enroll in college full-time, file the FAFSA, maintain Pell Grant eligibility with some remaining unmet need, and make academic progress toward a degree. Academic progress requirements are also part of Pell Grant eligibility, as defined by each college in terms of both courses completed and grades (Schudde & Scott-Clayton, 2016).

### 2.2 Potential effects of the WSG

Both economic reasoning and empirical evidence imply that the WSG funds could impact academic performance in several ways. As students made decisions about whether to stay enrolled, the WSG could have induced longer full-time enrollment at in-state institutions, by reducing the price of this specific pathway through college. The size of the population at this margin depends on the distribution of preferences and financial constraints across students, as formalized in Bettinger (2004). The university students receiving the WSG, as studied in Goldrick-Rab (2016), had higher levels of on-time degree completion, demonstrating that for a significant margin of students, the WSG was transformational.

Given that students had already enrolled, the WSG was intended not to attract students to college but to buffer against financial shocks that could lead to dropout. These unexpected shocks were important: in a survey of some WSG-eligible students in their first semester at two-year colleges, 34% said they were having more trouble affording college than they had expected. Financial aid was an important buffer, as 59% said they received no help from parents in paying for their education. If students had used online net price calculators to prepare for college, the living cost estimates they would have seen for Wisconsin two-year colleges were consistently underestimated relative to more objective measures from federal data, as shown by Kelchen, Goldrick-Rab, and Hosch (2017).

Whether or not additional financial aid reduced dropout, it could have changed students’ choices prior to dropping out. University students who were offered the WSG appeared to shift enrollment behavior to maintain eligibility for the aid, including maintaining a full credit load (Kinsley & Goldrick-Rab, 2015). If students with additional financial resources chose to work less in favor of more study time, this could have manifested itself in a higher rate of credit accumulation or better grades. There was room for reduction in work hours: in the same survey, 76% of students reported earning income from jobs during college. In a subset of the first cohort of WSG recipients at both universities and two-year colleges, Broton, Goldrick-Rab, and Benson (2016) found that students selected to receive the grant reported fewer work hours, and worked shifts that tended to be scheduled at times more conducive to success in classes.

The grant could also increase movement to universities by incentivizing those transfers, or could reduce transfers to other two-year colleges by meeting students’ needs at their initial institution. Bahr (2009) documented that lateral transfer between two-year colleges is quite common. Since transfers can delay degree completion, reducing transfer is potentially a positive impact of the WSG.

Even without affecting enrollment and work decisions, the additional grant aid could have induced students to borrow less, and leave college with lower amounts of student debt. Borrowing is relatively uncommon at most community colleges, and the majority of students in the WSG target population do not borrow. However a significant minority take student loans that could potentially be reduced, a key objective of the Fund (Fund for Wisconsin Scholars, 2008).

In spite of all these potential positives, effects of the WSG may have been hindered because it came without additional supports and imposed a small administrative burden which stymied some students. Among other things, the lack of structure around the grant led to a lack of salience: when the WSG recipient group was surveyed about which programs were part of their aid package, just 25% correctly included WSG in their choices, compared to 86% accuracy for the Pell Grant. WSG students were at least aware of the uncertainty they faced: just 13% of those surveyed agreed with the statement that “students receive the same amount of financial aid for every year they are in school.”

### 2.3 Research agenda

Given the different barriers to completion faced by community college students relative to university students who were the focus of earlier studies, we expect to find potentially different effects of WSG aid. To investigate these possibilities, we first estimate the effects of the WSG offer on students’ financial aid packages. The aid could potentially supplant other grants or cause students to reduce borrowing. Second, we estimate the effect of the WSG offer on short-term persistence. We measure credits attempted and completed, grades, and continued enrollment to the second year. Third, we extend the horizon, among a subset of students we can observe for three years, and measure the effect the WSG offer on persistence, degree completion, and transfer.

It is an open question for whom the marginal dollar of financial aid has its greatest impact. The Pell Grant measures the need for aid using an index called the Expected Family Contribution or EFC. The EFC takes into account household income, assets, and family structure, and it ranged from 0 to 5,273 among Pell Grant recipients during the period of our study. The Pell Grant offsets the EFC directly for these students. Therefore students with a higher EFC will have lower amounts of Pell Grant aid. They may not have sufficient resources to cover the additional out-of-pocket expense, if their positive EFC does not accurately capture what they can “contribute.” At the same time, students with a zero EFC cannot receive any more Pell Grant aid. Some of them can really “contribute” even less than an EFC of zero would imply, and thus might benefit from more aid.

We explore whether effects of the WSG differ between the zero-EFC group and positive-EFC group. The WSG design gives a unique ability to test for effects of additional financial aid in the zero-EFC group which is deemed the most needy, and for whom other eligibility cutoffs rarely bind. Effects of the university arm of the WSG on bachelor's degree attainment were generally no larger among students with lower out-of-pocket costs, which are associated with a lower EFC (Goldrick-Rab et al., 2016).

We also explore the adverse effects of a WSG offer that did not result in additional aid. We hypothesize that the offer of aid establishes a new baseline for students, and the later non-receipt is experienced as a loss (Kahneman, Knetsch, & Thaler, 1991). This up-and-down is not experienced by comparable students who were not randomly selected to receive a WSG offer.

To answer all these questions, we make use of the random assignment of WSG offers within a selected pool of students. We compare the outcomes of the WSG group to the outcomes of the remaining control group, and we attribute differences on average to the WSG intervention. After estimating treatment effects, we explore whether the WSG had different impacts on different subgroups of students. The fact that WSG offers are randomized allows for comparative estimates of effects across groups that can be identified at baseline.

### 2.4 Research design

We estimate regressions of the form:

(1) $$Y_{i}=\rho WSG_{i}+{X^{\prime }}_{i}\beta +{C^{\prime }}_{i}\gamma +{ɛ}_{i}$$

where *Y_i_* is some outcome for student *i*. Selection to receive a WSG offer, indicated by *WSG_i_*, varies randomly within the sample, making the residual ɛ_*i*_ uncorrelated with *WSG_i_* and providing unbiased estimates of the intent-to-treat effect *ρ*. Since eligible students are scattered all over the state among larger classes of college students, we do not expect there to be spillover effects of the WSG treatment across students in the sample. Therefore only the student's own assignment to the WSG is included, not the assignment of his or her peers.

We condition on a vector of cohort-by-system fixed effects ***C***_***i***_. These categories served as randomization blocks. To add precision, we condition on a vector of baseline characteristics ***X***_***i***_ describing household finances and family background, which may be related to outcomes. Throughout this study we use linear probability models for binary outcomes.

We test for impact heterogeneity by estimating Eq. (1) in subgroups. First, to test whether aid is more impactful for students with fewer family financial resources (but more government aid) we estimate effects within the zero-EFC group and compare estimates to the positive-EFC group.

Second, we explore the circumstances that led many students not to take up the WSG. We observe at baseline some of the WSG eligibility criteria: Wisconsin residency, age, full-time status, whether EFC is within the Pell range, and first-time enrollment status as reported on the FAFSA. Failing any one of these criteria leads us to classify students into a “predicted ineligible” group. Crucially, this prediction can be made just as easily in the control group. We then estimate Eq. (1) in the predicted ineligible group, to test for impacts of a grant offer that was unlikely to result in additional grant aid.

Our predicted eligibility accounts for a large proportion of the lack of WSG take-up. Among the group selected to treatment, the predicted eligible group had a 46 percentage-point higher likelihood of receiving WSG aid, jumping to 76% from 32%. Clearly there are still students who received aid in the predicted ineligible group and vice versa. Fitting a more flexible model of the propensity to receive WSG aid in the treatment group, using eligibility and other observables, does not yield markedly better explanatory power. It is also subject to missing data on initial WSG receipt in the WTCS sample, which comes from a different data source than the administrative records of aid at UW Colleges. We therefore choose to make use of our knowledge about program requirements to identify the predicted ineligible group rather than model post-randomization behavior.

## 3 Data and sample

This section places Wisconsin schools in the national context, then describes data collection, sampling, and baseline equivalence of the randomized groups.

### 3.1 Wisconsin setting

The importance of two-year colleges and two-year degrees varies widely across states. Wisconsin's two-year college enrollment is in the middle, ranking 27th (36th) out of 50 states in the percent of undergraduates (full-time undergraduates) enrolled in two-year public colleges (Ma & Baum, 2016). Wisconsin lags national averages in the fraction of the state workforce holding two-year liberal arts degrees (4.8% versus 6.3% nationally) and the wages earned by holders of two-year liberal arts degrees ($15.41 per hour versus $16.65 nationally) (COWS 2014). However for more career-oriented two-year degrees, these same figures in Wisconsin are above national averages (11.4% of the workforce versus 4.7% nationally, earning $18.29 versus $17.02 nationally) (COWS 2014).

Wisconsin two-year colleges belong to two distinct systems with campuses across the state. The state public university system provides liberal arts degrees and preparation to transfer to universities, via the University of Wisconsin Colleges (UW Colleges). UW Colleges is one institution consisting of 13 branch campuses and an online program. The technical college system is separate. The Wisconsin Technical College System (WTCS) consists of 16 colleges with 49 total branch campuses. WTCS focuses on preparing students for careers, though five of the colleges also include liberal arts and university transfer in their missions.

The two systems differ from each other and from national averages in a few key ways. Table 1 compares the two systems to each other and to the roughly 1,000 other two-year public colleges in the nation during the period of our study. The group of students beginning degree programs full-time (a requirement for the WSG) was a small fraction of total enrollment at both Wisconsin systems and nationally, though full-time degree-seeking enrollment was more common at UW Colleges. Over 40% of the students at both systems took out federal student loans, rates well above the national average of 20%. The rate of poverty, as indicated by Pell Grant eligibility, was lower at both systems (25% and 32%) than the national average of 45%.

**Table 1:** Characteristics of public two-year colleges and their students, 2008–09 school year.

	UW Colleges	WTCS	All public two-year
*Students*			
Enrollment	13,082	5,725	4,355
Under age 25 (%)	91.2	70.6	73.1
First-time, full-time, degree/certificate-seeking (%)	28.0	10.3	15.2
Of first-time, full-time, deg./certif. (%)			
Receiving Pell Grants	25.0	32.1	44.6
Receiving federal loans	41.0	46.8	19.7
Of those receiving federal financial aid (%)			
Living off-campus with family	60.0	41.3	48.2
Living off-campus not with family	33.3	56.5	43.3
*Prices*			
For full-time, off-campus, in-state ($)			
Tuition and required fees	4,584	3,265	2,799
Percent covered by WSG of $1,800 (%)	39.3	55.1	
Books and supplies	860	1,154	1,152
Total cost with family	9,464	7,326	7,502
Net price for federal aid recipients ($)			
Family income $0–30k	4,566	7,068	5,798
Percent covered by WSG of $1,800 (%)	39.4	25.5	
Family income $30k-48k	6,586	8,355	6,891
Percent covered by WSG of $1,800 (%)	27.3	21.5	
*Outcomes*			
Of first-time, full-time, deg./certif. receiving aid (%)			
Retention to fall 2009	58.0	62.1	59.1
Completed degree in 150% of normal time	20.9	35.5	26.8
Transferred out by end of 2010–11	37.9	21.8	19.7

Tuition setting, state financial aid, and official estimates of living costs all operate separately for the two systems, leading to some differences in measured prices. Considering tuition and required fees, the WSG covered 55% at WTCS versus 39% at UW Colleges. Considering net price for the lowest income group, WSG covered 26% at WTCS versus 39% at UW Colleges. The relative shift in percent covered at WTCS comes from the fact that less financial aid is typically available at technical colleges, leading to higher net prices even though tuition is lower.

Turning to outcomes, retention to the third semester was similar across both systems and the national sample, near 60%. Graduation rates differed widely between the two systems. At UW Colleges, 36% of students earned a degree within 150% of a normal time frame (three years for two-year associate degrees). At WTCS just 21% of students graduated in 150% of normal time (which could be shorter than three years for technical certificates). The national average was in between at 27%. UW Colleges students transferred out at a rate of 38%, far more often than WTCS students beginning at transfer-focused colleges (22%) or the national average (20%).

### 3.2 Study sample

This study uses administrative data for multiple cohorts of students who entered the WSG randomization pool. These data are well-suited to our inquiry as they provide accurate measures of key outcomes over time, as well as detailed student characteristics at baseline. The sources of data were the UW Colleges, WTCS, the Fund for Wisconsin Scholars, and the Wisconsin Higher Educational Aids Board.

**Fig. 1:**
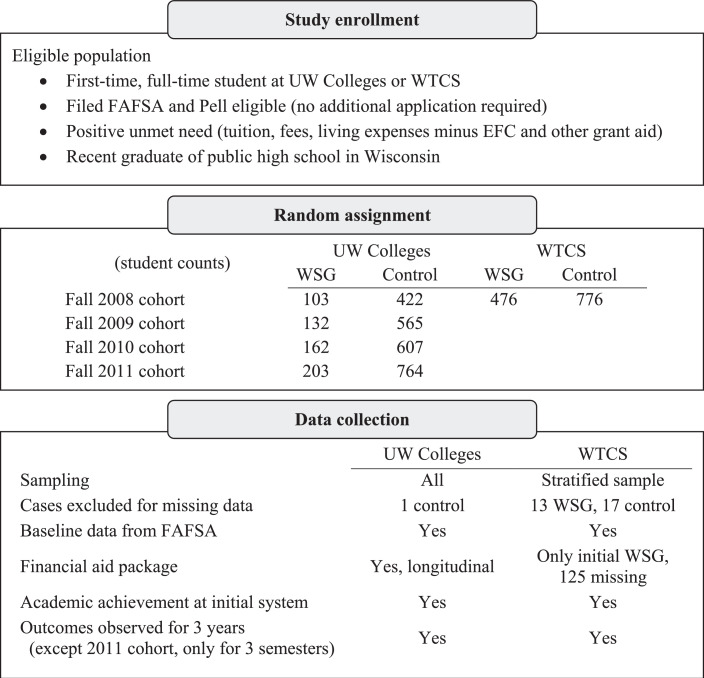
Flow diagram.

Fig. 1 lays out how students were sampled and how data were collected. Data from UW Colleges included all students in the WSG group and all students in the control group, from cohorts entering in fall 2008 through fall 2011. The window of observation includes school years 2008–09 through 2012–13. Data from WTCS included all students in the WSG group as well as a representative sample of the control group for the cohort entering in fall 2008, with a window of observation including school years 2008–09 through 2010–11. Thus we observe all of these students’ first year and continuation into a second, as well as the 2008, 2009, and 2010 cohorts’ first three years, corresponding to at least 150% of the normal time to earn a degree at UW Colleges or WTCS.

The WTCS administrative data come from a larger data collection effort on the fall 2008 cohort that included surveys and interviews, as described in Goldrick-Rab (2016). This effort limited the control group to a subsample, and employed a stratified random sample by college with different sampling rates across colleges. We use post-stratification weights so that the analysis sample is representative of the randomization pool (Lohr, 2009). Because of the sampling, standard errors are calculated by Taylor series linearization. Results do not differ substantially when estimated without weights, or when estimated with Huber-White robust standard errors.

In all there were 4,179 students in our study, 1,063 of whom were offered the WSG. For all students in the sample, we observe baseline characteristics from the FAFSA, and we observe enrollment, credits, grades, and degrees earned, as well as when and where students transfer. Detailed financial aid data are available semester-by-semester, but only for UW Colleges students. For all other analyses besides effects on financial aid, we group the systems together and control for fixed effects that capture average differences in outcomes across systems. The sample size limits statistical power to a minimum detectable effect size of 0.1 standard deviations of the outcome variable. In the case of persistence to a second year of college, that effect size corresponds to roughly five percentage points, or 2.7 percentage points per $1,000 in aid offered, an effect smaller than reported in earlier studies (Deming & Dynarski, 2010; Page and Scott-Clayton, 2016).

Overall there was very little missing data in these administrative records. Missing data either at baseline or for outcomes caused a weighted 0.8% rate of attrition in the control group and a 1.6% rate in the WSG group. As Fig. 1 indicates, most of the missing data are in the fall 2008 cohort at WTCS. The rates of missing data are not statistically significantly different in the WSG and control groups. Because randomization was executed properly and attrition is minimal, we expect this study to meet the standards of the federal What Works Clearinghouse without reservations (What Works Clearinghouse, 2017).

There are two main limitations of the data. First, we cannot report on effects of the WSG on student debt at WTCS. The reason for this limitation is that financial aid data are not centrally stored across colleges in the system and were too costly to collect. We have a measure of initial receipt of the WSG, that is observable for about three quarters of the treatment group, and comes directly from the Fund for Wisconsin Scholars. Second, student outcomes are limited to attainment within the student's starting system, with only the first destination of transfer recorded. The implications of the limitation on post-transfer outcomes are that for the 42% of students who do transfer, we cannot report on graduation at their destination institution. The reason for this limitation is that our data sources were the systems themselves, and not a broader source such as the National Student Clearinghouse.

A student's ultimate graduation rate at any college may be of greater interest than their graduation at the initial institution. The difference between this broader graduation effect and the one we measure depends on the effects of WSG on transfer, who is induced to transfer (or not) by additional grant aid, and if that group has different potential outcomes at their starting institution versus more broadly. If students who were on the margin of not succeeding at their first institution are induced to remain there longer because their costs are lowered, then we count their graduation outcomes but miss the graduation outcomes of their control counterparts who did transfer. If such students are trading away potentially better success at transfer institutions for lowered prices at the initial institution, then our measure of effects on graduation rates will be overstated relative to the effect on broader graduation rates.

Angrist et al., 2014, Angrist et al., 2016 and Denning et al. (2017) found that effects in the long-term may differ from effects in the short-term. Data collection on these samples will continue, so that we can also investigate whether this is true in the context of the WSG.

### 3.3 Baseline characteristics

To describe the sample, we report baseline characteristics in Table 2. WSG's targeting to young adult students from low-income families is evident. The average student is 18 or 19 years old, and 84% of the sample is considered financially dependent on their parents for financial aid purposes (meaning they have not married, had children, or served in the military). Seven percent of dependent students have parents with zero income. Over half of students work for pay in the year before they start college. With generally low levels of income when positive ($32,500 among parents), these household incomes translate into about half of students receiving the lowest possible EFC of 0. Race/ethnicity was observable in the UW Colleges sample only, and is not shown in the table. The vast majority (81%) of UW Colleges students were white, with 9% Asian, 4% Hispanic, 3% African American, and 1% Native American.

**Table 2:** Baseline equivalence of student characteristics across WSG and control groups.

	Control mean	WSG diff.	(SE)
Female (%)	58.5	−0.4	(1.9)
Age	19.5	0.1	(0.2)
Parent completed college (%)	38.9	−0.1	(1.9)
No parent with HS degree (%)	11.5	−0.2	(1.3)
Dependent for FAFSA (%)	83.7	1.1	(1.5)
Among dependent students			
Parent AGI is zero (%)	7.3	−0.1	(1.2)
Parent AGI if positive (current $)	31,885	1,732	(1,257)
Student AGI is zero (%)	39.7	2.5	(1.8)
Student AGI if positive (current $)	7,936	−687	(533)
EFC is zero (%)	47.4	0.6	(1.9)
EFC if positive (current $)	2,282	447	(233)

Table 2 reports the results of estimating Eq. (1) above, conditioning only on the cohort and system, with each baseline characteristic as the outcome, as an assessment of whether randomization established equivalent groups on average. The WSG group appears to have slightly higher EFCs, conditional on a positive EFC, significant at the 10% level. This and other differences are small in magnitude, and none are statistically significant at conventional levels. In the following section, we estimate Eq. (1) with these characteristics as covariates, to assess impacts on financial and educational choices.

## 4 Impacts of the Wisconsin Scholars Grant

This section reports regression results to answer our research questions. In all cases we report the mean of the outcome variable within the control group, then the estimated difference between the WSG group mean and the control group mean, adjusted for baseline covariates: indicators for cohort and initial system, which served as randomization blocks, as well as indicators for gender, mother's level of education, father's level of education, and initial dependency status, and continuous measures of age and initial EFC.

### 4.1 Impacts on financial aid

Changing a student's financial aid package was the initial, proximal effect of the WSG. Table 3 documents these changes at UW Colleges. In the control group, students received an average of $3,366 in grants. The net price after grant aid for a semester, as shown in Table 1, was roughly $2,300 to $3,300 for students in this income range. To help cover this, 45% of students borrowed, with an unconditional average of $886 in federal loans. Just 10% of students received work-study aid, and unconditional earnings from work-study were just $37.

**Table 3:** Effect of the WSG offer on initial financial aid at UW Colleges.

	Control mean	WSG diff.	(SE)
WSG (current $)	3	664	(16)
Other grants (current $)	3,366	−57	(52)
Took federal loans (%)	45	−1	(2)
Subsidized federal loan (unconditional, current $)	693	−40	(35)
Unsubsidized federal loan (unconditional, current $)	192	3	(19)
Received federal work study (%)	10	−1	(1)
Federal work study (uncondtitonal, current $)	37	2	(7)

The average student offered the WSG received $663 in WSG aid during the first semester, reflecting that many students received no aid. There was not a significant difference between the groups in terms of other grants received. This reflects the WSG policy that the grant not displace other grant aid, as well as that most grant aid received by these students is formulaic from the federal and state government, and therefore could not be reduced in response to a WSG offer.

Table 4 shows more detail on non-receipt of WSG aid in the group selected to treatment. At WTCS, using our best measure of initial receipt that nonetheless has some missing data, just 63% of the first cohort received WSG aid after selection. At UW Colleges, using administrative data on financial aid disbursement, the figure is 74% as reflected in the unconditional average from Table 3. Some but not all of non-receipt and non-renewal can be explained by observable eligibility factors of full-time enrollment, Pell Grant eligibility, and academic progress. We proxy for academic progress with an indicator for maintaining a cumulative GPA of 2.0 or higher. The rate of receipt among these observably eligible students at UW Colleges still falls from an initial 83% to 68% at the end of three years. Thus some students lose access to WSG because of unobserved factors: they do not have positive unmet need to cover, or they fail to reapply for the grant after losing eligibility or stopping out.

**Table 4:** Receipt of WSG among selected group.

	All	Enrolled full-time with Pell, above 2.0 GPA
Receiving WSG at WTCS (%)		
Semester 1	62.7	67.1
Receiving WSG at UW Colleges (%)		
Semester 1	74.1	82.7
Semester 2	66.6	84.5
Semester 3	38.9	81.3
Semester 4	34.0	78.8
Semester 5	10.4	66.2
Semester 6	4.3	68.3
Total WSG received ($)	2,063	

Over six semesters at UW Colleges, the average student offered the WSG received $2,063 from the program out of a potential $5,400 if enrolled for all six semesters. About a quarter of the WSG group did transfer to another WSG-eligible institution during this time, making this figure a potential underestimate of what they received from WSG in total. However enrolling at a WSG-eligible institution does not guarantee continued receipt.

### 4.2 Short-term impacts

Turning to educational outcomes, retention rates for community college students are often low, and this sample was no exception. Just 58% of students returned for a second year of college. The WSG aimed to change that, and Table 5 reports the results. The WSG effect on third-semester enrollment is nearly zero, with a relatively tight confidence interval. Studies of financial aid often report the effect per $1,000 offered (Deming & Dynarski, 2010). Scaling the effect of the grant for comparability across studies, a 95% confidence interval rules out impacts larger than 2.3 percentage points in likelihood of persistence per $1,000 in aid offered.

**Table 5:** Short-term effects of the WSG offer.

	Control mean	WSG diff.	(SE)
Semester 1			
Credits attempted	13.77	0.03	(0.09)
Credits earned	10.75	0.15	(0.19)
GPA	2.31	0.04	(0.04)
Semester 2			
Enrolled (%)	86.3	1.7	(1.3)
Enrolled full-time (%)	75.0	3.7	(1.7)
Credits attempted	11.57	0.31	(0.21)
Credits earned	8.94	0.02	(0.24)
Cumulative GPA above 2.0 (%)	63.6	0.0	(1.8)
Semester 3			
Enrolled (%)	58.4	0.5	(1.9)
Enrolled full-time (%)	48.4	2.9	(1.9)

The WSG also does not appear to increase grades or credit attainment while students are enrolled. Looking at control group outcomes in the first year, a GPA of 2.0 is often used as a benchmark for Satisfactory Academic Progress, and 65% of students met that requirement. Three quarters of students stayed enrolled full-time in the second semester. This resulted in 8.9 credits earned on average during the second semester. We do estimate a statistically significant increase of 3.7 percentage points in the rate of staying enrolled full-time in the second semester. There is also a positive point estimate for staying enrolled full-time in the third semester, suggesting that among the students remaining enrolled, the WSG may have induced more full-time enrollment to continue receiving the grant.

### 4.3 Longer-term impacts

Early stopouts or switches to part-time enrollment are common at two-year colleges, and do not necessarily mean that a student will not earn a degree. However, the lack of robust impacts of the WSG on short-term outcomes suggests that downstream impacts are unlikely. Table 6 shows longer-term outcomes for the fall 2008, 2009, and 2010 cohorts. There is a marginally statistically significant increase in the number of full-time semesters enrolled. The control group enrolled for 2.85 out of a possible 6, while the WSG group enrolls for 0.13 more, an increase of 5%.

**Table 6:** Effects of the WSG offer over three years.

	Control mean	WSG diff.	(SE)
Continued enrollment (%)			
Semester 2	86.7	1.3	(1.5)
Semester 3	59.0	0.01	(2.2)
Semester 4	50.9	0.8	(2.2)
Semester 5	27.9	0.8	(2.0)
Semester 6	23.1	−0.9	(1.9)
Degree through 4 sem. (%)	19.6	−0.5	(1.9)
Through 6 semesters			
Semesters enrolled	3.47	0.02	(0.07)
Full-time semesters enrolled	2.85	0.13	(0.07)
Credits earned	36.31	0.62	(1.04)
Cumulative GPA	2.23	0.00	(0.05)
Degree earned (%)	30.0	−1.0	(2.1)
Any transfer (%)	42.3	−3.9	(2.0)
Transfer to University of Wisc. (%)	22.6	−1.4	(1.4)
Transfer to two-year public (%)	6.5	−2.5	(1.2)
Transfer to for-profit (%)	2.8	0.2	(0.8)
Total debt among UW Colleges ($)	3,011	−245	(205)

Two years after beginning college, just 20% of students had completed a degree, and 28% continued to enroll for a third year. The remainder had stopped out or dropped out. Three years in, the average student had accumulated 36 credits, and 30% of students had completed a degree. There is no evidence of a positive impact of the WSG on these outcomes. With 95% confidence we can rule out impacts on three-year degree completion larger than 1.7 percentage points per $1,000 in aid offered.

Many students (42%) transferred at some point in their first three years after starting college. Most went to public universities in Wisconsin (23%). However 7% transferred to another two-year public college, and 3% transferred to a for-profit institution. Here the WSG had large and statistically significant effects: the WSG group was 2.5 percentage points less likely to transfer laterally to another two-year public college. Most of this effect was localized in students’ first year of college, where the rate of transfer in the WSG group was less than half that in the control group. As noted above, reducing tranfer could lead to faster attainment of degrees, but we do not have strong evidence that it did in this case.

### 4.4 Heterogeneous impacts

Given generally null estimates for treatment effects, heterogeneity in impacts would have to come from offsetting positive and negative impacts. We test for this kind of heterogeneity by EFC and predicted eligibility.

As noted earlier, EFC is the measure of financial need used by many financial aid programs. Because it captures socioeconomic status, the EFC tends to be positively correlated with student success. Since need-based aid is often targeted based on the EFC, it is difficult to discern how the effects of aid vary by underlying factors of socioeconomic status that are confounded with the EFC and aid amounts. In our setting we take advantage of random assignment to estimate the impacts of the WSG at varying levels of the EFC. The results appear in Table 7. For short-term persistence, the WSG did appear to have offsetting effects by EFC group, with small decreases in credits earned and continued enrollment for positive-EFC students, and small increases for zero-EFC students. Similar trends are present for longer-term measures, suggesting the WSG was potentially more impactful for students with lower EFCs. Though they received more aid at baseline from state and federal governments, the students in the zero-EFC group still appear to have had financial needs to meet using WSG aid. However when testing for differences in these coefficients, none of the differences are significant at conventional levels.

**Table 7:** Heterogeneous effects of the WSG offer by EFC.

	EFC zero		EFC positive	
	WSG	(SE)	WSG	(SE)
Short-term effects				
Enrolled in semester 3 (%)	1.9	(2.8)	−0.7	(2.6)
Credits earned in first year	1.02	(0.57)	−0.57	(0.58)
Effects over three years				
Semesters enrolled	0.04	(0.11)	0.005	(0.09)
Full-time semesters enrolled	0.17	(0.10)	0.10	(0.09)
Credits earned	1.62	(1.46)	−0.05	(1.47)
Degree earned (%)	2.6	(2.9)	−3.8	(2.8)
Cumulative GPA	0.07	(0.07)	−0.05	(0.06)
Any transfer (%)	−3.5	(2.9)	−3.9	(2.7)

Offering the WSG to apparently ineligible students (most of whom never received it) had a statistically significant and large negative impact on their odds of returning for a second year of college, reducing it by 9.3 percentage points. These estimates appear in Table 8. This reduction in persistence also shows up in negative point estimates for longer-term effects, but the magnitudes are smaller and standard errors are larger. However the short-term estimate provides evidence of a negative effect of an unsubstantiated offer of financial aid.

**Table 8:** Heterogeneous effects of the WSG by predicted eligibility.

	Predicted ineligible		Predicted eligible	
	WSG	(SE)	WSG	(SE)
Short-term effects				
Enrolled in semester 3 (%)	−9.7	(4.6)	3.2	(2.1)
Credits earned in first year	0.35	(1.00)	0.03	(0.44)
Longer-term effects				
Semesters enrolled	−0.26	(0.16)	0.11	(0.08)
Full-time semesters enrolled	0.06	(0.15)	0.16	(0.07)
Credits earned	−1.79	(2.04)	1.34	(1.21)
Degree earned (%)	−0.9	(4.6)	−1.2	(2.3)
Cumulative GPA	−0.05	(0.11)	0.004	(0.05)
Any transfer (%)	−1.1	(4.2)	−4.6	(2.2)

## 5 Discussion and conclusion

This study evaluated the effects of a supplement to the Pell Grant, offered to a randomly selected group of students at two-year colleges in Wisconsin. The grant's requirements and implementation led to many of its intended recipients not receiving it or retaining it over time. Still, students offered the grant received an average of just over $2,000 in additional support over a three-year period. This support does not appear to have improved their odds of persisting in college or completing degrees on average.

The WSG example showed that even small added complexity, for students and administrators, can get in the way of students receiving their aid. The WSG case is especially informative for policymaking by states and private foundations that do not have the ability to change pricing directly, have secondhand access to eligibility criteria and financial aid packaging, and can only provide small price adjustments relative to overall student costs. The experience of the WSG has makes clear that it can be surprisingly hard to give away money and have it make an impact.

The surprise element of providing aid after enrollment, here arising from a brand new and randomized grant program, is common to other more established programs and to prices in general. The Pell Grant formula changes nearly every year, and the program has also made larger changes such as shifting lifetime limits and yearly credit limits (Mabel, 2017, Liu, 2017). Changes in financial aid can also come from the actions of students who miss performance cutoffs (Carruthers and Özek 2016, Schudde and Scott-Clayton 2016) or who fail to reapply for aid (Bird & Castleman, 2016). Public college tuition changes come from shifts in geographic discount policies (Denning, 2017b), from tuition freezes and caps at the state level (Deming & Walters, 2017), and from institution-level changes (Hemelt & Marcotte, 2011). The general consensus emerging from studies of net price changes is that student enrollment is responsive to price when it is salient.

Whether uncertainty itself deters student persistence is less well known, because reductions in uncertainty typically cannot be disentangled from other factors. Guaranteed tuition plans in some states reduce uncertainty about tuition changes, but they shift financing burdens onto early-career students both directly, and indirectly through other school or state-level policy changes that tend to accompany these plans (Delaney and Kearney, 2015, Delaney et al., 2016). A nationwide shift to earlier FAFSA filing was meant to give students information about aid earlier, but it also changed the basis of aid eligibility by shifting it back one year (FSA, 2015).

Our study has the feature of randomly assigned complexity and uncertainty. We show that when this complexity is not accompanied by aid, it can lead to adverse effects. This finding is consistent with a large body of work on the take-up of social benefits (Currie, 2004). The application and enrollment process imposes costs that are often large enough to apparently outweigh the benefits of the aid itself. Recent work shows that these hassle costs can be overcome using timely informational interventions (Barr and Turner, 2018, Bhargava and Manoli, 2015, Page et al., 2018). We find that students offered the WSG enrolled in more full-time semesters at their initial institution, suggesting that some students were informed enough to understand how to remain eligible for the WSG and willing to shift behavior to remain eligible. However these shifts do not appear to have led to overall increases in attainment.

Though the WSG shares some similarities with an expansion to the Pell Grant for particular students, the answer to whether expansions to the Pell Grant ultimately increased community college degree attainment during the late 2000 recession is still unclear. Any complete analysis must expand beyond students enrolling directly out of high school, the focus of this study, to include older adults who enrolled in large numbers during the down labor market, and who represent an important part of the community college landscape. A more complete understanding of the effectiveness of financial aid will inform how aid fits into the array of public and private efforts to ameliorate the problem of community college dropout.

## Data access

The data for this study were accessed under agreements between the authors and the data providers, for the purposes of this research only. Therefore they cannot be made available publicly. Researchers interested in requesting the same administrative records may contact the following offices: Office of Policy Analysis and Research, University of Wisconsin System www.wisconsin.edu/offices/office-of-academic-and-student-affairs/office-of-policy-analysis-research, Office of Information Technology, Wisconsin Technical College System wtcsystem.edu/about-us/governance/policy-overview/administrative-services/information-technology, Fund for Wisconsin Scholars ffws.org, Higher Educational Aids Board www.heab.state.wi.us.

In Section 2.2 we report calculations from the survey data described in Goldrick-Rab (2016). These survey data are not required to replicate the study's results, and only provide context for the study. The microdata from these survey responses cannot be made available publicly, because of confidentiality agreements with survey respondents. Researchers with questions about these data may contact the corresponding author Drew M. Anderson drew@rand.org.

## Declaration of interest

The authors have no interests to disclose.
